# In silico analysis of promoter regions to identify regulatory elements in TetR family transcriptional regulatory genes of *Mycobacterium colombiense* CECT 3035

**DOI:** 10.1186/s43141-022-00331-6

**Published:** 2022-03-31

**Authors:** Feyissa Hamde, Hunduma Dinka, Mohammed Naimuddin

**Affiliations:** Department of Applied Biology, School of Applied Natural Science, Adama Science and Technology University, P.O. Box 1888, Adama, Ethiopia

**Keywords:** *M*. *colombiense*, Transcription start site, Promoter, Motif, CpG islands, Antibiotic resistance

## Abstract

**Background:**

*Mycobacterium colombiense* is an acid-fast, non-motile, rod-shaped mycobacterium confirmed to cause respiratory disease and disseminated infection in immune-compromised patients, and lymphadenopathy in immune-competent children. It has virulence mechanisms that allow them to adapt, survive, replicate, and produce diseases in the host. To tackle the diseases caused by *M*. *colombiense*, understanding of the regulation mechanisms of its genes is important. This paper, therefore, analyzes transcription start sites, promoter regions, motifs, transcription factors, and CpG islands in TetR family transcriptional regulatory (TFTR) genes of *M*. *colombiense* CECT 3035 using neural network promoter prediction, MEME, TOMTOM algorithms, and evolutionary analysis with the help of MEGA-X.

**Results:**

The analysis of 22 protein coding TFTR genes of *M*. *colombiense* CECT 3035 showed that 86.36% and 13.64% of the gene sequences had one and two TSSs, respectively. Using MEME, we identified five motifs (MTF1, MTF2, MTF3, MTF4, and MTF5) and MTF1 was revealed as the common promoter motif for 100% TFTR genes of *M*. *colombiense* CECT 3035 which may serve as binding site for transcription factors that shared a minimum homology of 95.45%. MTF1 was compared to the registered prokaryotic motifs and found to match with 15 of them. MTF1 serves as the binding site mainly for AraC, LexA, and Bacterial histone-like protein families. Other protein families such as MATP, RR, σ-70 factor, TetR, LytTR, LuxR, and NAP also appear to be the binding candidates for MTF1. These families are known to have functions in virulence mechanisms, metabolism, quorum sensing, cell division, and antibiotic resistance. Furthermore, it was found that TFTR genes of *M*. *colombiense* CECT 3035 have many CpG islands with several fragments in their CpG islands. Molecular evolutionary genetic analysis showed close relationship among the genes.

**Conclusion:**

We believe these findings will provide a better understanding of the regulation of TFTR genes in *M*. *colombiense* CECT 3035 involved in vital processes such as cell division, pathogenesis, and drug resistance and are likely to provide insights for drug development important to tackle the diseases caused by this mycobacterium. We believe this is the first report of in silico analyses of the transcriptional regulation of *M*. *colombiense* TFTR genes.

## Background

*Mycobacterium colombiense* is an acid-fast, non-motile, rod-shaped mycobacterium that belongs to the *Mycobacterium avium* complex (MAC) [1]. MAC contains clinically important non-tuberculous mycobacteria (NTM) and is the second largest medical complex in the *Mycobacterium* genus after the *Mycobacterium tuberculosis* complex [2]. MAC comprises species that include *M*. *colombiense*, *M*. *avium*, *M*. *intracellulare*, *M*. *chimaera*, *M*. *marseillense*, *M*. *timonense*, *M*. *boucherdurhonense*, *M*. *vulneris*, *M*. *arosiense*, four subspecies of *M*. *avium*, and “MAC-other” species [3]. NTM are believed to be natural inhabitants of the environment, found as saprophytes, commensals, and symbionts in the ecosystem. Since their clinical relevance was unknown, these bacteria have been neglected for many years as they have always been recognized as just environmental contaminants or colonizers [4]. Although, they are not considered as a public health problem, their importance is increasing due to their frequent association with immune-suppression, especially in HIV/AIDS patients, which is highly fatal [5].

NTM are generally acquired from the environment via ingestion, inhalation, and dermal contact [6]. They are opportunistic pathogens that cause lymphadenitis, lung infections, skin, and soft tissue infections mostly affecting patients with preexisting pulmonary disease such as chronic obstructive pulmonary disease or tuberculosis (TB), or those with systemic impairment of immunity (i.e., patients with HIV infection, leukemia, and those using immunosuppressive drugs) [1, 7]. There are more than 150 non-tuberculous mycobacterial species listed in public databases and about a third of them have been implicated in diseases of humans [4]. NTM has been observed for 100 years, but the trend of increasing prevalence of NTM is of great concern for clinicians as well as microbiologists. In some areas, NTM-associated disease is more abundant than previously believed and is a quietly unfolding disease epidemic, even overtaking TB prevalence which results in an increase in the medical costs [8]. NTM are an important cause of morbidity and mortality in the progressive lung diseases [9] where they are also important pathogens because of their high level of antitubercular drug resistance [10].

Among NTM, *M*. *colombiense* has been confirmed to cause respiratory disease and disseminated infection in immune-compromised HIV patients, as well as lymphadenopathy in immune-competent children. Nevertheless, very little is known about the molecular mechanisms that underlie *M*. *colombiense* gene expression regulation that play a great role in infection and pathogenesis [11]. Mycobateria are known to display differential drug susceptibility and strong drug resistance to several antibiotics by various mechanisms [12]. Understanding the regulatory pathways involved in drug resistance would aid the drug development process against this pathogen and possibly NTM [13]. TetR family transcription regulators (TFTRs) play a significant role in conferring antibiotic resistance and also control expression of biosynthesis of antibiotics, pathogenicity, biofilm formation, quorum sensing, cytokinesis, morphogenesis, osmotic stress, and various metabolic pathways [14, 15]. A recent report indicated that TFTRs represent the most abundant class of regulators in mycobacteria [16]. However, there are no reports about the regulatory analysis of TFTRs of *M. colombiense* CECT 3035 in silico predictions of transcriptome data could provide key information on the molecular details of regulatory mechanisms including promoter sequences, type of sigma factors associated to the RNA polymerase (RNAP) involved in the initiation of transcription, as well as other regulatory elements [17]. The objective of this study was therefore, to analyze transcription start site (TSS), promoter regions, transcription factors (TF), and cytosine-phosphate-guanine (CpG) islands in TFTR genes of *M*. *colombiense* CECT 3035 to gain insights into the regulation of gene expression*.* We also discuss about the role of drug resistance and possible directions for drug development.

## Materials and methods

### Identification of transcription start site and promoter region

Twenty two encoding genome sequences of TFTR genes of *M*. *colombiense* CECT 3035 starting with prokaryotic start codons (ATG, GTG, and TTG) were identified from National Center for Biotechnology Information (NCBI) database. First, the sequences with start codons were identified and used to determine their TSS. To find TSS, 1 kb sequences upstream of prokaryotic start codon were excised from each gene sequence. In most of the TFTR genes of *M*. *colombiense* CECT 3035, since the TSS regions are confined beyond 1 kb upstream of a start codon, an additional 1 kb, 2 kb, or more sequences from prokaryotic start codons were excised from each gene sequence. Promoter regions for the anticipated gene in *M*. *colombiense* were defined as 1 kb length upstream of each TSSs. For this purpose, the sequences were prepared in the Fasta format and entered into neural network promoter prediction (NNPP version 2.2) tool. NNPP tool was set with minimum standard predictive score (between 0 and 1) cutoff value of 0.8 for prokaryotes [18]. In order to have more accurate prediction value, the highest value of prediction score was considered for regions containing more than one TSS on NNPP output.

### Identification of motifs and transcription factors

To identify motifs and transcription factors, 22 sequence encoding genes of *M*. *colombiense* were downloaded from GenBank of NCBI database in their Fasta format. After the collection of genes, the whole gene promoters were identified for each gene using NNPP algorithm to find possible transcription promoters in prokaryotic organisms. All identified *M*. *colombiense* promoter sequences were analyzed using Multiple Em for Motif Elicitation (MEME version 5.3.3) search tool/web server hosted by the National Biomedical Computation Resource to look for motifs and transcription factors that regulate the expression of genes [19]. In addition to motif and transcription factor discovery, MEME is also important in carrying out motif scanning, motif enrichment, motif comparison, and gene regulation [20]. From the optional inputs in MEME, Classic mode (for motif discovery), DNA (for sequence alphabet), zero or one occurrence per sequence (for site distribution) were kept as a default, while five (for the number of motifs MEME should find) were set prior to start searching. Since zero occurrence per sequence or one occurrence per sequence models are sufficient for most motif finding [21], zero or one occurrence per sequence was applied for motif distribution. MEME outputs the result as MEME HTML (High Pretext Markup Programming Language), MEME XML, MEME text output, MAST HTML, MAST XML, and MAST text. We used MEME HTML to discover motifs and motif locations. The discovered motifs were displayed as a request and the motif locations were displayed in the form of block diagrams.

Following MEME results, one of the discovered motifs with the smallest *e* value was forwarded to other web-based program (TOMTOM) that compares one or more motifs against a database of known motifs for further investigation. The output of TOMTOM includes LOGOS representing the alignment of two motifs, the *p* value and *q* value (a measure of false discovery rate) of the match and links back to the parent motif database for more detailed information about the target motif. TOMTOM shows the query motif closely resembles the binding motif (transcription factor) in the set of *M. colombiense* CECT 3035 gene promoter regions [22].

### Identification of CpG islands

To find the CpG islands in TFTR genes of *M*. *colombiense* CECT 3035, both promoter regions and body regions were used. Accordingly, two algorithms were used. The first algorithm was Takai and Jones’ stringent algorithm—CpG island finder (Database of CpG Islands–http://dbcat.cgm.ntu.edu.tw/). This algorithm was used since it outperforms the others in excluding the short interspersed elements and can identify CpGs that are more likely associated with the 5′ regions of genes [23]. The second was CLC searching genomics Workbench ver. 3.6.5 (http://clcbio.com, CLC Bio, Aarhus, Denmark). It was used for searching CpG islands using the restriction enzyme *MspI* cutting sites (fragment sizes between 40 and 220 bp).

### Phylogenetic tree

Phylogenetic tree was constructed using molecular evolutionary genetics analysis X (MEGA-X version 10.2.6) using neighbor-joining method [24] with the use of aligned protein sequences from TFTR genes of *M*. *colombiense* CECT 3035. The tree was drawn to scale with branch lengths showing the evolutionary distances those infer phylogenetic tree. The evolutionary distances were computed using the maximum composite likelihood method [25] and are in the units of the number of base substitutions per site. All ambiguous positions were removed for each sequence pair. Evolutionary analyses were conducted in MEGA X [26]. Bootstrap tests were also performed to estimate the phylogeny of the sequences. All ambiguous positions were removed for each sequence pair (pairwise deletion option). There were a total of 1000 positions in the final dataset.

## Results

### Transcription start sites identification

From the 22 encoding genome sequences of TFTR genes of *M*. *colombiense* CECT 3035, 17 (77.27%), 4 (18.18%), and 1 (4.55%) genes start with ATG, GTG, and TTG, respectively. TSS of all the 22 genes were identified using NNPP. Accordingly, the highest prediction score was considered to determine the promoter regions for genes containing more than one transcription start sites. The results show that most genes (19 genes, 86.36%) contain single TSS and only 3 genes (13.64%) contain two TSSs using predictive score at the cutoff value of 0.8. Looking at their distance from the start codon, the farthest gene was found 11,105 bp away from the start codon at 92% predictive score and the closest gene was found 24 bp away from the start codon at 83% predictive score (Table 1).

**Table 1:** Identified TSSs, predictive score value, and their distances from start codon

Gene name	Gene ID	Number of TSS identified	Predictive score at cut value of 0.8	Distance from start codon
MCOL_RS11660	31527733	1	0.94	2390
MCOL_RS11090	31527620	2	0.91, 0.92	− 11,928, − 11,105
MCOL_RS08825	31527172	1	0.99	− 943
MCOL_RS08145	31527038	1	0.89	1150
MCOL_RS06375	31526691	1	0.82	3946
MCOL_RS04605	31526350	1	0.89	478
MCOL_RS03660	31526168	1	0.83	24
MCOL_RS02905	31526017	1	0.93	5711
MCOL_RS02170	31525872	1	0.91	− 2877
MCOL_RS26110	31530593	2	0.97, 0.99	1780, 952
MCOL_RS07580	31526928	1	0.93	− 52
MCOL_RS05990	31526617	1	0.9	7888
MCOL_RS23260	31530032	1	0.82	6063
MCOL_RS22650	31529910	1	0.85	− 677
MCOL_RS17010	31528795	1	0.82	10425
MCOL_RS03070	31526050	1	0.95	− 1426
MCOL_RS04305	31526295	1	0.9	− 2778
MCOL_RS22305	31529842	2	0.82, 0.91	4568, 4044
MCOL_RS20235	31529434	1	0.90	1645
MCOL_RS25615	31530496	1	0.90	1468
MCOL_RS15085	31528411	1	0.85	7727
MCOL_RS13375	31528074	1	0.82	113

### Common motifs and transcription factors

**Fig. 1:**
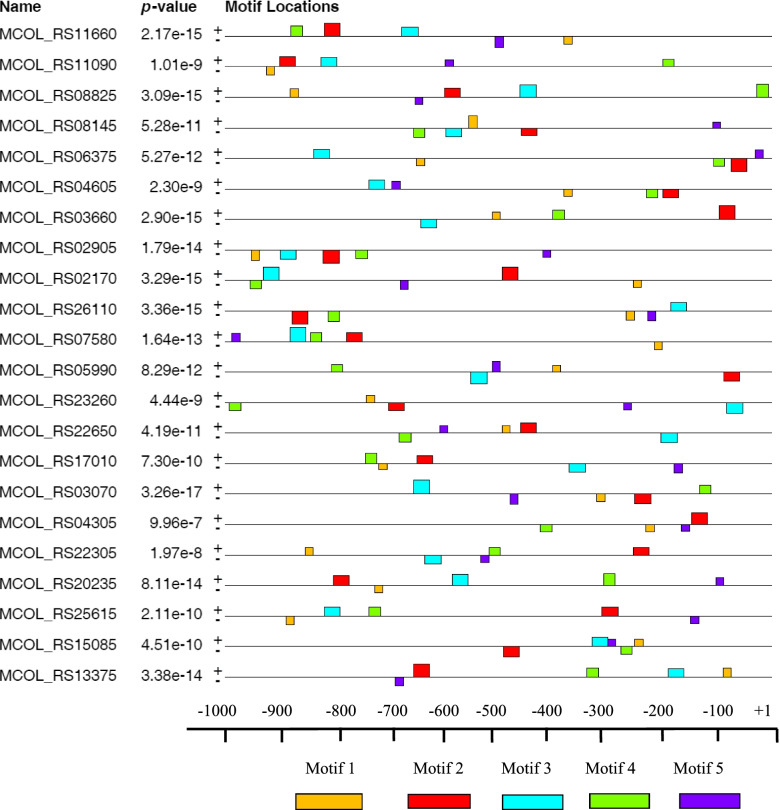
Positions of motifs relative to TSSs. The nucleotide positions are specified at the bottom of the graph from + 1 (beginning of TSSs) to the upstream 1 kb (− 1 kb) bp

After the identification of TSS, promoter regions were identified for each gene and loaded to MEME. Accordingly, significant motifs in the input sequence set was searched using MEME through web server and the *E* value which is the probability of finding well conserved pattern in random sequences. MEME output revealed five motifs (MTF1, MTF2, MTF3, MTF4, and MTF5). MTF1 was found to be the common motif for 100% with the lowest *E* value (1.1e-013) and a motif width of 29 bp which serve as binding sites for transcription factors sharing a minimum of (95.45%) (Table 2). MTF1 was found to serve as binding sites for transcription factors in the expression and regulation of the genes. Of the total 108 motifs, slightly higher distributions were found in positive strands (56) than negative strands (52) of TFTR genes of *M*. *colombiense* CECT 3035. The location and distribution of the motifs were found between − 994 and − 9 bp of the transcription start sites (Fig. 1).

**Table 2:** Identified common motifs in gene promoter regions and number of binding sites

Discovered motif	Number (%) of CECT 3035 promoters containing the motifs	*E* value^a^	Motif width	Total no. of binding sites
MTF 1	22 (100%)	1.1e-013	29	22
MTF 2	21 (95.45%)	3.0e-007	29	21
MTF 3	22 (100%)	6.8e-006	21	22
MTF 4	21 (95.45%)	1.5e-003	15	21
MTF 5	22 (100%)	1.8e-001	15	22

*MTF* motif

^a^Probability of finding an equally well conserved motif in random sequences

**Fig. 2:**
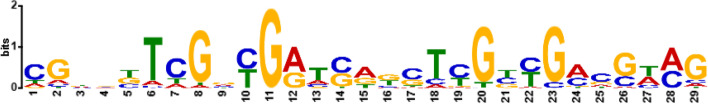
Sequence logos for the identified common promoter motif, MTF1 of *M*. *colombiense* CECT 3035 genes. The analysis was carried out using the MEME Suite

To analyze the information content, sequence logos for the common promoter motif (MTF1) was generated by MEME (Fig. 2). This resulted in different characters of motif alignment columns, where the height of the letter represents how frequently that nucleotide is expected to be observed at the defined position.

Furthermore, MTF1 was compared to the registered motifs in publicly available databases so as to check if there are similarities to known regulatory motifs using TOMTOM web application. In a similar manner, TOMTOM provides LOGOS that represent the alignment of two motifs and a numeric score for the match between two motifs. The output from TOMTOM also links back to the parent motif database for detailed information on the biological functions of the matched motif. The results show that MTF1 matched with 15 out of 84 known motifs found in Prokaryotic DNA databases. Looking at the ratio, MTF1 matched with 4 AraC families, 2 LexA families, 2 Bacterial histone-like protein families, and one family of each SLC45 (MATP) family, RR family, σ-70 factor family, TetR family, LytTR family, LuxR family, and NAP family. The matched motifs and their biological roles are shown in Table 3. Based on the functions, we categorized the TFs into different groups. The majority of the matched TFs (7/15) are found to be involved in pathogenesis by the generation of different virulence factors and antibiotic resistance. We grouped other TFs by functions related to Information storage and replication (2/15), metabolism (3/15), and stress survival (3/15).

**Table 3:** Transcription factor families binding to MTF1 motif of the promoter regions from prokaryotic database and their roles

TF families	Candidate transcription factors and species	*E* value	Function
AraC family	VqsM, *P*. *aeruginosa*	2.46e-02	Control the production of virulence factors and quorum-sensing signaling molecules
AraC *family*	AmrZ, *P*. *aeruginosa*	3.37e-01	Associated with biofilms and quorum sensing
SLC45 (MATP) family	MatP, *E*. *coli*	3.51e-01	Involved in cross-linking of DNA in the Ter MD and linking the chromosome to the divisome together with ZapA and ZapB
RR family	CtrA, *C*. *crescentus*	6.12e-01	Regulate morphogenesis, DNA replication initiation, DNA methylation, cell division, and cell wall metabolism.
σ-70 factor family	PvdS, *P*. *aeruginosa*	1.70e+00	Activate transcription of genes for the biosynthesis or the uptake of siderophores.
TetR family	RutR, *E*. *coli*	1.95e+00	Regulate genes (rutABCDEFG operon) involved in degradation and synthesis of pyrimidine, degradation of purines, glutamine supply and pH homeostasis.
LytTR *family*	AlgR, *P*. *aeruginosa*	3.05e+00	Control alginate production, type IV pilus function and virulence
NAP family	EspR, *M*. *tuberculosis*	4.24e+00	controls the virulence of Mtb by regulating expression of EspA
Bacterial histone-like proteins	IHF, *P*. *putida*	4.46e+00	Downregulated genes encoding ribosomal proteins, the alpha subunit of RNA polymerase and components of the ATP synthase.
AraC family	ArgR, *P*. *aeruginosa*	4.91e+00	Have major role in the control of certain biosynthetic and catabolic arginine genes
LexA families	LexA, *S*. *aureus*	6.28e+00	Govern Salt overly sensitive (SOS) response
Bacterial histone-like proteins	IHF, *P*. *putida*	7.34e+00	Essential for xyl gene expression from the TOL plasmid and for the biodegradation of benzyl alcohol
LuxR protein family	LasR, *P*. *aeruginosa*	7.52e+00	Involved in transcription, enhances exotoxin A production, plays role in pathogenesis
LexA families	LexA, *C. glutamicum*	8.07e+00	Represses a number of genes involved in the response to DNA damage
AraC family	HrpX, *X. oryzae*	8.36e+00	Regulation of virulence and motility required for pilus assembly

### Determination of CpG islands

To further explore the regulatory elements that are involved in TFTR genes of *M*. *colombiense* CECT 3035, CpG islands were investigated in its promoter and gene body regions using CpG island finder (http://dbcat.cgm.ntu.edu.tw/) and CLC searching genomics Workbench ver. 3.6.5 (http://clcbio.com, CLC bio, Aarhus, Denmark). Accordingly, only 4 (MCOL_RS08145, MCOL_RS05990, MCOL_RS17010, and MCOL_RS04305) of 22 genes of TFTR genes of *M*. *colombiense* CECT 3035 lack CpG islands in their promoter regions with GC content greater than 50% in all genes as parameter set of Obs/Exp greater than 0.65. Similarly, only 1 (MCOL_RS22650) of the 22 genes lack CpG islands in their body regions while all the remaining genes contain one possible CpG island with GC content greater than 61.1%.

On the other hand, digestion of TFTR genes of *M*. *colombiense* CECT 3035 using CLC genomics workbench ver 3.6.1 with *MspI* restriction enzyme showed a single CpG island in one gene, and all the remaining 21 genes have multiple CpG islands in their promoter regions (Table 4).

**Table 4:** Determination of *MspI* sites and fragment sizes for promoter regions

Names of corresponding promoter regions	Nucleotide positions of *MspI* sites	Fragment sizes (between 40 and 220 bp)
Pro-MCOL_RS11660	Multiple (17, 64, 163, 223, 336, 476, 484, 692, 706, 742, 827, 838, 886, 937)	47, 99, 60, 113, 140, 208, 85, 48, 51
Pro-MCOL_RS11090	Multiple (108, 130, 446, 504, 586, 656, 665, 707, 839, 867, 995)	58, 82, 70, 42, 132, 128
Pro-MCOL_RS08825	Multiple (21, 143, 163, 258, 343, 362, 379, 439, 653, 691, 752, 768, 953, 995)	122, 95, 85, 60, 214, 61, 185, 42
Pro-MCOL_RS08145	Multiple (27, 107, 206, 269, 388, 475, 499, 727, 764, 774, 849, 885, 962)	80, 99, 63, 119, 87, 75, 77
Pro-MCOL_RS06375	Multiple (50, 175, 239, 299, 428, 460, 557, 591, 595, 618, 735, 739, 750, 769, 866, 982, 994)	125, 64, 60, 129, 97, 117, 97, 116
Pro-MCOL_RS04605	Multiple (126, 157, 180, 329, 574, 613, 634, 837, 907, 985)	149, 203, 70, 78
Pro-MCOL_RS03660	Multiple (12, 116, 173, 213, 253, 426, 473, 540, 648, 654, 707, 857)	104, 57, 40, 173, 47, 67, 108, 53, 150
Pro-MCOL_RS02905	Multiple (21, 92, 137, 251, 610, 818, 828, 931)	71, 45, 114, 208, 103
Pro-MCOL_RS02170	Multiple (57, 204, 224, 261, 308, 416, 435, 471, 563, 592, 604, 733, 874, 965)	147, 47, 108, 92, 129, 141, 91
Pro-MCOL_RS26110	Multiple (14, 140, 168, 241, 545, 571, 653, 665, 868)	126, 73, 82, 203
Pro-MCOL_RS07580	Multiple (66, 165, 204, 255, 273, 461, 489, 516, 594, 642, 724, 758)	99, 51, 188, 78, 48, 82
Pro-MCOL_RS05990	Multiple (295, 312, 327, 347, 906, 968, 985)	62
Pro-MCOL_RS23260	Multiple (91, 97, 115, 295, 302, 391, 450, 550, 557, 627, 687, 709, 745, 821, 953)	180, 89, 59, 100, 70, 60, 76, 132
Pro-MCOL_RS22650	Multiple (5, 79, 86, 225, 239, 318, 330, 348, 435, 457, 553, 624, 640, 716, 765, 844, 888)	74, 139, 79, 87, 96, 71, 76, 49, 44
Pro-MCOL_RS17010	Multiple (5, 23, 33, 40, 130, 195, 247, 403, 543, 585, 680, 688, 716, 724, 851, 910)	90, 65, 52, 156, 140, 42, 95, 127, 59
Pro-MCOL_RS03070	Multiple (80, 158, 170, 209, 222, 341, 388, 415, 509, 577, 604, 706, 785, 923, 955, 965)	78, 119, 47, 94, 68, 102, 79, 138
Pro-MCOL_RS04305	Multiple (11, 149, 165, 170, 409, 804, 886, 906, 922, 964)	138, 82, 42
Pro-MCOL_RS22305	Multiple (175, 236, 248, 302, 554, 616, 636, 729, 782, 946)	61, 54, 62, 93, 53, 164
Pro-MCOL_RS20235	Multiple (10, 14, 20, 47, 380, 402, 571, 641, 820, 979)	169, 70, 179, 159
Pro-MCOL_RS25615	Multiple (0, 22, 92, 142, 148, 184, 254, 290, 310, 377, 851, 876)	70, 50, 67
Pro-MCOL_RS15085	Multiple (13, 18, 105, 127, 200, 223, 306, 369, 497, 553, 565, 992)	87, 73, 83, 63, 128, 56
Pro-MCOL_RS13375	Multiple (58, 83, 227, 242, 295, 329, 393, 422, 553, 618, 652, 861, 884, 893)	144, 53, 64, 131, 65, 209

Likewise, digestion of the body regions of TFTR genes of *M*. *colombiense* CECT 3035 by *MspI* restriction enzyme showed 1 gene lacking CpG islands, 5 genes with single CpG islands, 4 genes with two CpG islands, and all the remaining 12 genes with multiple CpG islands (Table 5).

**Table 5:** *MspI* cutting sites and fragment sizes for gene body regions

Name of corresponding body region	Nucleotide positions of *MspI* sites	Fragment sizes
MCOL_RS11660	Multiple (23, 41, 53, 127, 152, 162, 189, 241, 374, 378, 432, 450, 517)	74, 52, 133, 54, 67
MCOL_RS11090	Multiple (124, 193, 231, 340)	69, 109
MCOL_RS08825	Multiple (9, 130, 155, 240, 361, 395, 529)	121, 85, 134
MCOL_RS08145	Multiple (130, 243, 360)	113, 117
MCOL_RS06375	Multiple (4, 35, 182, 540)	147
MCOL_RS04605	Multiple (121, 267, 542)	146
MCOL_RS03660	Multiple (109, 152, 227, 448, 557, 566)	43, 75, 109
MCOL_RS02905	Multiple (98, 275, 286, 303)	177
MCOL_RS02170	Multiple (306, 431, 464, 565)	125, 101
MCOL_RS26110	Multiple (288, 420, 446, 458)	132
MCOL_RS07580	Multiple (74, 133, 158, 200, 459, 554, 586, 616)	59, 42, 95
MCOL_RS05990	Multiple (185, 274, 539)	89
MCOL_RS23260	Multiple (4, 118, 214, 225, 263, 276, 280, 320, 369, 519, 541)	114, 96, 40, 49, 150
MCOL_RS22650	Multiple (16, 23, 39, 43, 261, 361, 442, 592, 603)	218, 55, 81, 150
MCOL_RS17010	Multiple (29, 231, 310, 388, 445, 524, 540)	202, 79, 78, 57, 79
MCOL_RS03070	Multiple (95, 189, 203, 292, 311, 440, 484, 608, 643, 652)	94, 89, 129, 44, 124
MCOL_RS04305	Multiple (20, 278, 377, 394, 485, 489)	99, 91
MCOL_RS22305	Multiple (243, 540)	-
MCOL_RS20235	Multiple (27, 52, 140, 149, 274, 382, 459, 493, 518, 558, 613, 624)	88, 125, 108, 77, 40, 55
MCOL_RS25615	Multiple (14, 113, 133, 387, 480, 607)	99, 93, 127
MCOL_RS15085	Multiple (68, 127, 246, 310, 367, 401, 459, 505, 529, 566)	59, 119, 64, 57, 58, 46
MCOL_RS13375	Multiple (49, 57, 106, 183, 291, 296, 396, 440, 466, 598, 604)	49, 77, 108, 100, 132

### Analysis of phylogenetic tree

**Fig. 3:**
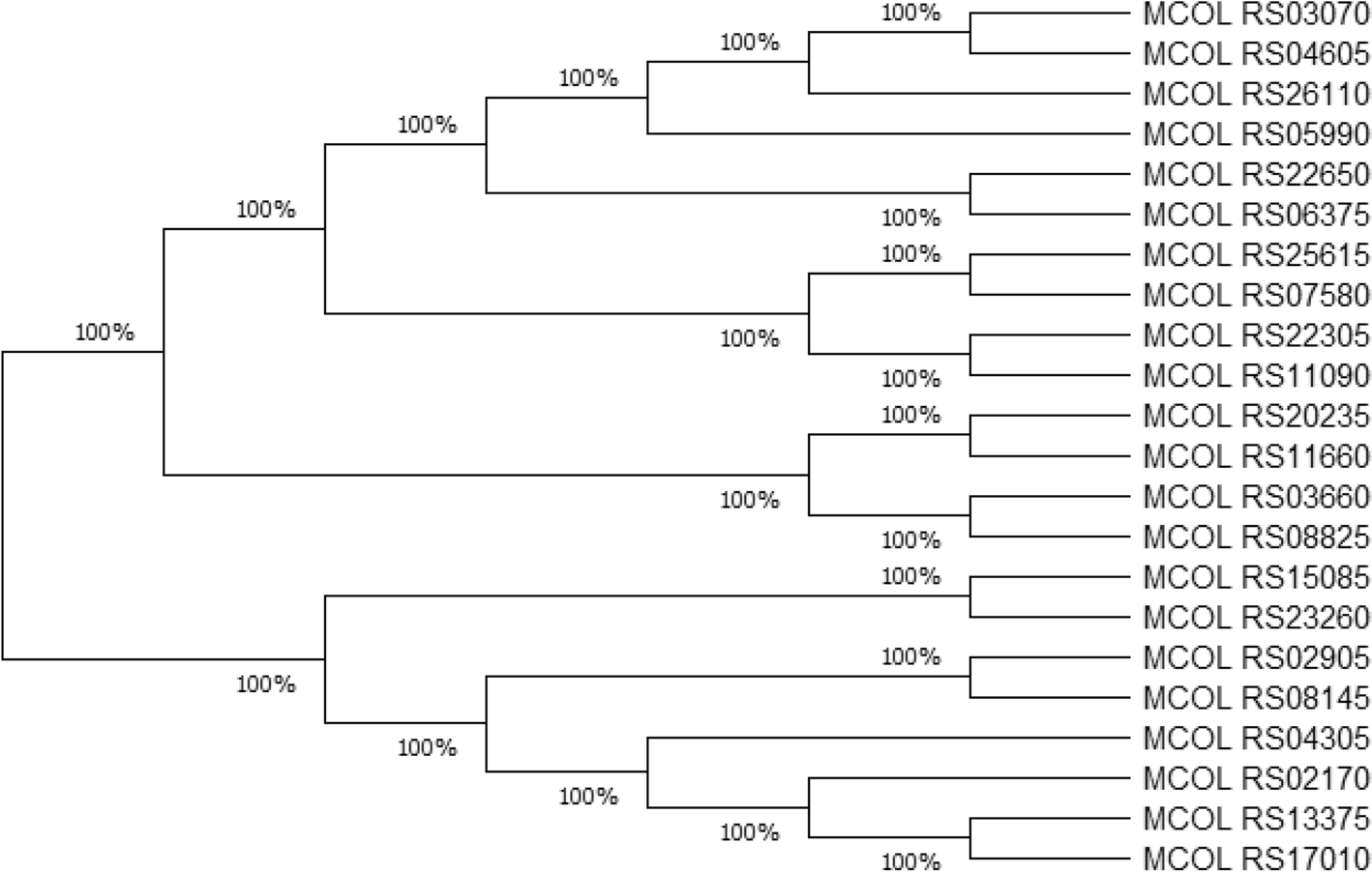
Phylogenetic tree of *M*. *colombiense* CECT 3035 genes using neighbor-joining method

In recent years, the purpose of phylogenetic trees was expanded to include understanding the relationships among the sequences without regard to the host species, inferring the functions of genes that have not been studied experimentally and elucidating mechanisms that lead to microbial outbreaks [27]. Here, a phylogenetic tree was constructed using the neighbor-joining method and minimum-evolution method of MEGA-X as shown in Fig. 3. Even though homologous evolutionary ancestor supported with 100% bootstrap was shown in the following phylogenetic tree, different closely related clusters and sister groups were observed.

## Discussion

The DNA sequences around TSSs are important for gene regulation in bacteria. Pinpointing of these TSS permits the identification of potential binding sites for transcriptional regulators those may inhibit or promote translation [28]. In this study, 86.36% and 13.64% genes were found to have a single and two TSSs, respectively. For those genes with two TSSs, TSS with a higher value was considered. This result is in agreement with Boutard et al. [29] where most genes were expressed from a single TSS. Identification of transcription start sites enables identification of promoter regions [30]. Hence, using the identified TSSs of each gene the promoter region was identified for every gene. The promoter element defines the DNA site directing the RNA polymerase for transcription initiation, and it is a crucial element to understand gene expression in bacteria [31]. Accurate prediction of promoters is fundamental for interpreting gene expression patterns, and for constructing and understanding genetic regulatory networks [32]. After the discovery of promoter regions for each gene, we used each promoter sequence to identify motifs and transcription factors using MEME. From the five identified motifs, motif 1 (MTF1) was found as the most common regulatory motif for the TFTR genes of *M*. *colombiense* CECT 3035 to regulate expression of genes (Table 2). The motif width of MTF1 was found to be 29 bp which is in agreement with a recent report which found a motif length of 27 bp represented DNA binding site for a TetR-dependent regulation of a drug efflux pump in *Mycobacterium abscessus* [33]. In addition, results of MEME also indicated the particular location and distributions of motifs to largely occur between − 974 bp and − 9 bp from transcription start site. This confirms the location of motif to be upstream, neighborhood of the TSS in corresponding with other transcription factors [34].

Additionally, the comparison of query motif (MTF1) with registered motifs in publicly available database of *M*. *colombiense* CECT 3035 genes using TOMTOM web application showed that MTF1 matched with 15 out of 84 known motifs found in prokaryotic DNA databases (Table 3). MTF1 matched with 4 AraC families, involved in pathogenesis by the production of virulence factors, dormancy survival and drug resistance by the formation of biofilms, cell-to-cell communication, and arginine metabolism; 2 LexA families, involved in survival by inducing SOS response upon DNA damage and salt stress management; 2 bacterial histone-like protein families, involved in metabolism of aromatic compounds and downregulation of genes for entering into stationary phase; and one family of each SLC45 (MATP) family, involved in replication; RR family, involved in cell cycle programs of chromosome replication and genetic transcription; σ-70 factor family, for virulence and mobilization of metals by siderophores; TetR family, for cell growth by pyrimidine catabolism; LytTR family, for virulence by control of alginate production and type IV pilus function; LuxR family, for pathogenesis by production of endotoxin A; and NAP family, controls the virulence of *M*. *tuberculosis* by regulating expression of *EspA*. These findings match with the functions of TFTR that play a significant role in conferring antibiotic resistance and also control the expression of biosynthesis of antibiotics, pathogenicity, biofilm formation, quorum sensing, cytokinesis, morphogenesis, osmotic stress, and various metabolic pathways [14, 15]. Formation of biofilms is an important strategy in bacteria for survival, pathogenesis, and antibiotic resistance [3, 35]. The presence of glycophospholipids on the outermost portions of the cell envelope enables formation of biofilms on the hyrdrophobic surfaces. Biofilms allow communication and exchange of materials between the closely associated cells and has been linked to confer antibiotic resistance [13, 35, 36]. Antibiotic resistance by biofilms is a complex process which has various modes of action such as the formation of barrier where the exopolysaccharide component greatly reduces permeability to antibiotics, detoxification mechanism which produces enzymes to disrupt or alter the structure of antibiotics that render them inactive, drug efflux pumps that reduces the intracellular concentration of antibiotics by transporting antibiotics outside of the cell, and drug sequestration where specific proteins prevent binding of antibiotics to the targets [35, 37, 38]. The potentially increased horizontal gene transfer between the closely interacting bacterial cells in the biofilms may also contribute to the spread of antibiotic resistance [37, 38]. Effective anitmicrobials can be designed based on this knowledge against bacterial biofilms [39]. Quorum sensing (QS) is an important cell-cell communication process that play significant roles in regulation of a variety of biological processes such as virulence gene expression, biofilm formation, drug efflux pumps, and plasmid transfer [37, 40]. In QS regulatory systems, microorganisms produce and release a diffusible autoinducer or QS signal to the surrounding environment, which accumulates along with bacterial growth and induces target gene transcriptional expression upon interaction with the respective signal receptor. In this study, we found MTF1 binds to two TFs, VqsM and LasR, from *P*. *aeruginosa* that have been reported to play a role in virulence and QS modulation that positively regulates the QS systems [40, 41]. QS signals could offer an important possible direction for the development of antimicrobials by the design of antagonists based on enzymes that can abolish the QS signals or QS inhibitors that can interfere with the signaling process. Based on the type of signals used by the microbes, i.e., conserved or unique signals, the choice of the design of broad-specificity or narrow-specificity antimicrobials could possibly be facilitated [37, 42]. Iron is an essential element required for microbial growth and virulence. Siderophore molecules (also called mycobactins) are sophisticated iron-acquisition systems to overcome iron deficiency imposed by the host defensive mechanism. These small molecules are secreted into the extracellular space, tightly bind available iron, and then are reinternalized with their bound iron through specific cell surface receptors [43]. Antimicrobial susceptibility with respect to iron metabolism in MAC has been shown to be dependent on mycobactins. Under iron-restricted conditions, the susceptibility to antibiotics such as ethambutol, isoniazid, and d-cycloserine that target cell wall synthesis increased [44]. In this study, MTF1 was found to match with the TF, PvdS from *P*. *aeruginosa* involved in the biosynthesis or the uptake of siderophores [45] and may be a potential target for the development of antimicrobials. The other matched TFs for virulence by various mechanisms include AlgR, EspR (regulates gene expression of EspA) and HrpX [2, 46, 47]. Interestingly, EspR has been found to be conserved in *M*. *tuberculosis* and *M*. *colombiense* [2]. One of the most important aspect of survival of living organisms is metabolism that determines growth or dormancy, several biosynthetic processes including DNA replication and division of cells. Several TFs were found to match with the binding motif MTF1 revealed in this study. RutR, belonging to TetR family, is involved in the regulation of degradation and synthesis of pyrimidines, degradation of purines, glutamine supply, and pH homeostasis by mechanisms that both stimulates and inhibits gene expression at different promoters [48]. ArgR has been found to play a major role in the control of certain biosynthetic and catabolic arginine genes [49]. Integration host factor (IHF) is known to be involved in a large number of cellular functions; however, it plays a major regulatory role during transition from exponential to stationary phase by controlling various cell surface-related functions and downregulating genes encoding ribosomal proteins, the alpha subunit of RNA polymerase, and components of ATP synthase. IHF also controls xylR, which is the master transcriptional factor of the TOL pathway for biodegradation of m-xylene [50]. Mycobacterial infections are known to be difficult to treat due to this switchover from growth to stationary phases. Cell division is a vital process for survival and propagation for living organisms. In this study, we have found two transcription factors MatP (*Escherichia coli*) and CtrA (*Caulobacter crescentus*) involved in cell division process taking part in mechanisms such as linking chromosome to the divisome along with ZapA and ZapB, initiation of DNA replication, morphogenesis, DNA methylation, and cell wall metabolism among other functions [51, 52]. In recent years, bacterial cell division has been recognized as a promising new direction for the discovery of antibiotics. Filamenting temperature-sensitive mutant Z (FtsZ) protein has emerged as a promising target for drug discovery. FtsZ is an essential and central protein that has the ability to organize into dynamic polymers at the cell membrane to form a “divisome.” Most cell division inhibitors act via FtsZ, either by interfering with GTPase activity or the assembly/disassembly of the Z-ring, as well as by destabilizing the structure of FtsZ [53]. LexA family transcription factor is involved in transcriptional repressor [54]. Therefore, targeting SOS response might play a central role in promoting survival and the evolution of resistance under antibiotic stress [55]. Identification and understanding of the transcriptional regulatory process by TFs revealed in this study could provide important insights into the development of antimicrobials against *M*. *colombiense* CECT 3035 and possibly other NTMs and open gates for further research.

CpG Island is a pattern that plays a crucial role in the analysis of genomes. It consist high-frequency of CpG dinucleotides [56]. CpG islands are DNA methylations regions in promoters known to regulate gene expression through transcriptional silencing of the corresponding gene. DNA methylation at CpG islands is crucial for gene expression and tissue-specific processes [57]. In the present study, an investigation of the CpG islands was performed for both promoter regions and body regions of *M*. *colombiense* CECT 3035 genes using CpG island finder and *MspI* restriction enzyme digestion. Only 5 of 22 genes of *M*. *colombiense* CECT 3035 genes lack CpG islands in their promoter regions and only 2 of them lack CpG islands in their body regions with GC content greater than 50% and 61.1% in promoter regions and body regions respectively, while all the rest (17 genes, 77.27% promoter gene and 20 genes, 90.91% body regions) contain one possible CpG island. On the other hand, digestion of the promoter regions of *M*. *colombiense* CECT 3035 genes with *MspI* showed 1 gene with single fragment and all the remaining 21 genes with multiple fragments in their promoter regions, whereas CpG islands of 1 genes lacking fragment, 5 genes contain single fragment, CpG islands of 4 genes contain two fragments, and the remaining 12 body region genes were found to contain multiple fragments, respectively. This result is comparable with a recent report with regard to digestion by *MspI* enzyme (the existence of several fragments (28/29) in promoter regions and several fragments (26/29) in body regions of *Herbaspirillum seropedicae* genes) [58]. This result implies that the promoter region of *M*. *colombiense* CECT 3035 genes have rich CpG islands that can play a crucial role in gene regulation.

The phylogenetic tree generated in this study showed 22 different branches representing different genes. The branching patterns of the tree indicated that a shared evolutionary history existed among the genes with 100% bootstrap. In addition, it is clear that there were different clusters and sister groups those may differ from each other due to base substitutions in the sequences. Hence, knowing these features can contribute significantly to our knowledge on molecular evolution, species phylogeny, and biotechnology [59, 60] which may help in tackling the spread of the bacteria. Furthermore, the close relatedness of the genes is also the characteristics of *M*. *colombiense* since study of DNA–DNA relatedness clearly differentiates *M*. *colombiense* as separate species within the MAC [61].

## Conclusion

*M*. *colombiense* is a member of MAC responsible to cause respiratory disease and disseminated infection in immune-compromised patients and lymphadenopathy in immune-competent children. Therefore, understanding of the mechanisms and its components that regulate gene expression is very important in order to tackle the infection of this mycobacterium. TFTRs are known to play diverse regulatory functions including antibiotic resistance, pathogenicity, biofilm formation, quorum sensing, cytokinesis, morphogenesis, osmotic stress, and various metabolic pathways. In this paper, transcription start site, promoter region, binding motifs, and CpG islands of TFTR genes of *M*. *colombiense* CECT 3035 were analyzed. Accordingly, TSSs of 22 genes were identified and five motifs were found to be shared by at least 95.45% genes of *M*. *colombiense* CECT 3035 promoter input sequences. Among the five motifs, MTF1 was identified as a common promoter motif shared by (100%) TFTR genes of *M*. *colombiense* CECT 3035 promoters. MTF1 was compared to the known Prokaryotic DNA motif databases and identified to match with 15 out of 84 known motifs. The matched TFs were found to be in good agreement with the regulatory functions of TFTRs and indicated good candidates for the development of antimicrobials including biofilms, quorum signals, siderophores, biosynthesis of cell wall, metabolic states, and cell division and may help to design a combination of therapeutic molecules. These findings are anticipated to provide knowledge for the discovery and development of antimicrobials and possibly next-generation antimicrobials against *M*. *colombiense* CECT 3035 and other NTMs as well. Furthermore, analysis of CpG islands showed the existence of a high frequency of CpG islands in both promoter and body regions of genes of *M*. *colombiense* CECT 3035 that can have epigenetic regulatory implications while molecular evolutionary genetic analysis showed close relationships among the genes.

## Data Availability

Data analyzed in current study were taken from NCBI database of CECT 3035 genes of *M*. *colombiense.*

## Abbreviations

MAC: *Mycobacterium avium* complex
NTM: Non-tuberculous mycobacteria
RNAP: RNA polymerase
TFs: transcription factors
TFTR: TetR family transcription regulator
CpG: Cytosine-phosphate-guanine
NCBI: National Center for Biotechnology Information
TSS: Transcription start site
NNPP: Neural network promoter prediction
MEME: Multiple Em for Motif Elicitation
MEGA-X: Molecular evolutionary genetics analysis X
MTF: Motif
